# Magnetoimpedance Response and Field Sensitivity in Stress-Annealed Co-Based Microwires for Sensor Applications

**DOI:** 10.3390/s20113227

**Published:** 2020-06-05

**Authors:** David González-Alonso, Lorena González-Legarreta, Paula Corte-León, Valentina Zhukova, Mihail Ipatov, Juan María Blanco, Arcady Zhukov

**Affiliations:** 1 Departmento CITIMAC, Facultad de Ciencias, Universidad de Cantabria, E-39005 Santander, Spain; david.gonzalezalonso@unican.es; 2 Departmento QUIPRE, Inorganic Chemistry-University of Cantabria, Nanomedice-IDIVAL, Avda. de Los Castros 46, 39005 Santander, Spain; 3 Departmento de Física de Materiales, Facultad de Químicas, Universidad del País Vasco/Euskal Herriko Unibersitatea, UPV/EHU, Paseo Manuel de Lardizabal, 3, 20018 San Sebastián, Spain; paula.corte@ehu.eus (P.C.-L.); valentina.zhukova@ehu.es (V.Z.); mihail.ipatov@ehu.es (M.I.); 4 Departmento de Física Aplicada, EIG, Universidad del País Vasco/Euskal Herriko Unibersitatea, UPV/EHU, 20018 San Sebastian, Spain; juanmaria.blanco@ehu.es; 5 IKERBASQUE, Basque Foundation for Science, 48011 Bilbao, Spain

**Keywords:** magnetoimpedance effect, skin effect, soft magnetic materials, field sensitivity, amorphous microwires

## Abstract

Amorphous soft magnetic microwires have attracted much attention in the area of sensor applications due to their excellent properties. In this work, we study the influence of annealing treatments (stress and conventional) in the giant magnetoimpedance (GMI) response and the field sensitivity of the soft magnetic Co_69.2_Fe_3.6_Ni_1_B_12.5_Si_11_Mo_1.5_C_1.2_ glass-coated microwires. Here we report a remarkable and simultaneous enhancement of GMI effect and field sensitivity. The highest sensitivity of 104%/Oe and the GMI response of 234% were achieved for 300 °C stress-annealed samples at 472 and 236 MPa, respectively. Additionally, we found that stress-annealed microwires exhibit a frequency dependence on maximal GMI response and field sensitivity. These findings are obtained by fine-tuning their magnetoeslastic anisotropies through stress-annealing treatments of as-prepared microwires at the proper temperature and axial applied stress upon annealing. We hope that the results presented here widen the scope of investigations for the future design of soft magnetic materials for sensor purposes.

## 1. Introduction

Over the last decades, magnetic sensors have captivated scientific attention for their technological findings in a broad scope of fields. These applications range from space research, military applications, security systems, high-density magnetic recording and biomedicine [1,2,3,4,5,6]. In this line, the miniaturization of sensors and technological devices opens new routes in the property-to-function conversion. Metal-based amorphous alloys [7,8] constitute the main family within magnetic sensors. After Panina et al. [7,8], Co-based rapidly quenched materials have become widely recognized magnetic materials for sensors due to their high-performance properties [9,10]. It should be highlighted that their excellent magnetic softness, exhibiting nearly zero magnetostriction constant value and a remarkably giant magnetoimpedance (GMI) response along with high field sensitivity, among other properties [7,11,12].

In this sense, giant magnetoimpedance (GMI) effect results is the most remarkable feature for sensor applications. This phenomenon consists in a significant change of the AC impedance in presence of a static dc-field [13]. GMI ratios up to 600%, at frequencies around 10 MHz [14] and sensitivities up to 10%/Am^−1^ are the maximum values reported to date for amorphous wires [15]. These properties make Co-based materials quite suitable candidates for GMI technology, such as magnetic field sensors integrated in complementary metal-oxide-semiconductor (CMOS) circuits [16], high sensitive magnetometers [17] or biomagnetic field detection [18,19].

Co-based glass-coated microwires are attracting scientific interest for their potential in the development of small-scale sensors based on their tunable magnetic properties. The Co-based microwires investigated in this work are fabricated by the Taylor-Ulitovsky technique [20,21]. The Taylor-Ulitovsky technique involving rapid solidification of metallic alloys inside the glass coating allows the preparation of long (up to 10 km) continuous composite metallic microwires coated by a flexible and insulating glass cover (typical thickness of 0.5–20 μm). At suitable fabrication conditions, a completely amorphous structure with no trace of crystalline phases can be obtained [20,21]. Magnetocrystalline anisotropy contribution is consequently negligible, while their magnetic properties are principally governed by the magnetoelastic interactions. Furthermore, the magnetoelastic interactions play a key role in the GMI response [15,21,22]. In fact, these interactions are determined by both the magnetostriction coefficient and internal stresses [23]. Apart from these contributions, classical electrodynamics gives the fundamental support to explain satisfactorily the GMI effect by considering the skin effect of a magnetic conductor [24]. Based on this assumption, the most relevant pre-requisite to achieve a giant MI response is to design amorphous microwires with significant circumferential magnetic permeability, which can be reached by fine-tuning their magnetic properties [25,26,27].

With respect to the magnetoelastic anisotropy of glass-coated microwires, the magnetostriction coefficient, λ_s_, is dictated by the chemical composition (at fixed internal stresses values which in turn are affected by the fabrication conditions) [28]. Thus, the replacement of Fe by Co atoms permits the adjustment of λ_s_–values from positive (typically λ_s_ ~40 × 10^−6^ for Fe–based microwires) to negative (about λ_s_ ~ −5 × 10^−6^ for Co–based microwires) values [28,29]. Accordingly, the vanishing λ_s_–values can be achieved in Co-Fe or Co-Mn amorphous alloys when the content of Co/Fe or Co/Mn is about 70/5 [28,29]. Moreover, for alloys with vanishing λ_s_–values the stresses influence can be relevant [28]. On the other hand, the internal stresses (value and distribution), which arise from the fabrication of glass-coated microwires, are the other factor affecting the magnetoelastic anisotropy [15,21,23]. The internal stresses, *σ_i_*, value can be caused by: i) the thermal expansion coefficient mismatch between metallic-core and the glass-coating; ii) the quenching stresses originated during the rapid solidification process; and iii) the drawing stresses [30,31]. According to most theoretical estimations, the internal stresses arising from the thermal expansion coefficient mismatch are expected to present the largest contribution [30,31].

In this regard, at a fixed composition and geometry (metallic nucleus diameter and glass-coating thickness) the magnetic anisotropy can be fine-tuned either by the internal stresses relaxation (usually by conventional annealing) or by inducing magnetic anisotropy [27,32]. However, it has been recently noticed that conventional annealing produces a considerable magnetic hardening in different Co-rich microwires with vanishing λ_s_-values [32,33]. Such magnetic hardening is a detriment to the GMI performance [27,32,33]. Nevertheless, we have recently observed that stress-annealing can be successfully employed to induce a transverse anisotropy and hence improve the GMI effect of magnetic microwires [27,32,33].

For those reasons, our aim is to identify the routes that allow the optimization of GMI performance at a fixed chemical composition and geometry (i.e., with fixed λ_s_ and *σ_i_* values). We expect that in the future the observed dependencies can be extended to various Co-rich microwires.

Accordingly, in this work, we have investigated the magnetic properties of Co-based amorphous microwires with a well-established chemical composition under stress-annealing conditions aiming to simultaneously enhance their GMI response and magnetic field sensitivity within the range 10 ≤ *f* ≤ 1000 MHz of intermediate frequencies.

## 2. Experimental Methods

The Co_69.2_Fe_3.6_Ni_1_B_12.5_Si_11_Mo_1.5_C_1.2_ microwire consists in a metallic nucleus of d = 22.8 µm diameter, surrounded by an outer glass-coating shell that results in D = 23.2 µm total diameter. The amorphous microwire has been fabricated using the Taylor-Ulitovsky technique, which is described elsewhere [20,21]. As in similar Co-based microwires [28], the sample presents a slightly negative magnetostriction coefficient *λ_S_* ≈ −1 × 10^−7^ [27]. This allows us to predict the magnetic softness in our sample and suggests the existence of a circumferential anisotropy along the microwire. This latter feature is the underlying ingredient to obtain remarkable MI response [24,34].

For proper modification to the magnetic properties of our sample, slices of the as-prepared microwire were subsequently heat-treated in a standard furnace by two different methods. In one method the annealing is performed with no applied stress, whereas in the second it is conducted under axial tensile stress. The tensile stress, *σ_m_*, was applied through a mechanical load attached to the end of the microwire and axially placed via the furnace nozzle, allowing stresses up to 472 MPa. The *σ_m_*-values are evaluated considering the different Young’s moduli of the metallic nucleus and the glass coating as recently described [33]. All these treatments were performed at selected temperatures, *T_ann_*, ranged from 200 to 400 °C and for 1 h duration, *t_ann_*. This annealing temperature range has been selected considering the onset of the crystallization process reported at ca. 490 °C for microwires of the same composition [35]. In fact, it has been described that the rising of the annealing temperature close to the crystallization point causes a deterioration of both mechanical and magnetic properties in Co-rich microwires [36,37]. Although two of the advantages of glass-coated microwires are their flexibility and insulating properties of the glass coating, an excess of temperature can damage the outer glass-coating.

Magnetic hysteresis loops (HLs) were measured using the fluxmetric method already described [33]. Microwire slices of 5 cm in length were placed inside a single layered pick-up coil, where a magnetic field was created by a 15 cm long solenoid. For better comparison of the treated microwires, HLs are represented as the normalized magnetization *M/M*_0_, where *M* is the measured magnetic moment at a given magnetic field, and *M*_0_ is the maximal magnetic moment obtained at the highest magnetic field amplitude *H_max_*.

Impedance measurements, Z, were carried out with a vector network analyzer (VNA) N5230A at room temperature. Microwires of 6 mm length were fixed to a micro-strip sample holder by tin soldering, and subsequently placed inside a long solenoid that creates a maximum homogeneous magnetic field, *H*, of 15 kA/m (ca. 189 Oe). Z-values are indirectly obtained in the intermediate frequency range 10–1000 MHz through the measurement of the reflection coefficient S_11_, using the following expression [34,38]:

(1) $$Z=Z_{0}\frac{{(1+S}_{11})}{{(1-\;S}_{11})}$$

where *Z*_0_ = 50 Ohm is the characteristic impedance of the coaxial line.

The MI response or GMI ratio, ΔZ/Z, is determined from Z-values, which are obtained for different magnetic fields measurements, and it is defined as:

(2) $$\Delta Z/Z=\frac{[Z\left(H\right){-Z(H}_{max})]}{{Z(H}_{max})}\times 100$$

where *H_max_* is the highest dc-magnetic field applied.

A distinctive feature of MI response to discriminate among magnetic sensor materials is the field sensitivity, *η*, which is calculated through:

(3) $$\eta =\frac{\partial \left(\frac{\Delta Z}{Z}\right)}{\partial H}$$

## 3. Results and Discussion

**Figure 1:**
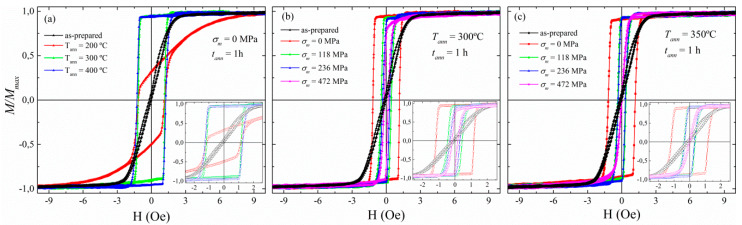
(**a**) Hysteresis loops (HLs) of the as-prepared and the annealed microwires performed at 200 °C, 300 °C, and 400 °C. Subfigures (**b**) and (**c**) show HLs of as-prepared and stress-annealed performed at *T_ann_* = 300 and 350 °C, respectively. The thermal treatments on the parent-microwire Co_69.2_Fe_3.6_Ni_1_B_12.5_Si_11_Mo_1.5_C_1.2_ were performed for 1h. In the insets, it is shown a magnification of the HLs to make clear the linear-to-rectangular evolution of the treated microwires.

**Figure 2:**
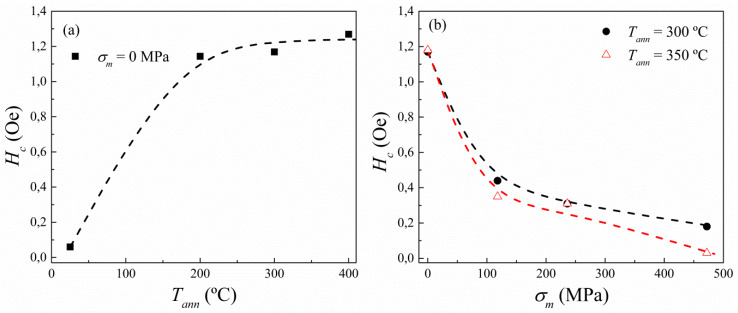
Temperature-annealing (**a**) and stress-annealing (**b**) dependence on the *H_c_* of Co_69.2_Fe_3.6_Ni_1_B_12.5_Si_11_Mo_1.5_C_1.2_ microwires for 1 h of thermal-treatment. Dashed lines are a guide to the eyes.

The MI response in amorphous ferromagnetic microwires can be enhanced by relaxing their inner stresses through a diversity of thermal treatments. In view of this, we present here the advantages of stress-annealing when optimising the magnetic properties of the Co_69.2_Fe_3.6_Ni_1_B_12.5_Si_11_Mo_1.5_C_1.2_ as-prepared microwire, in contrast to conventional annealing. Figure 1a shows the HLs of the as-prepared, along with the annealed microwires performed at selected temperatures *T_ann_* below its crystallization point (~550 °C, [35]). In the inset of Figure 1a, it is observed a magnetic hardening after 1h annealing. Particularly, in Figure 2a we depict the effect of conventional annealing by increasing the *H_c_* from ca. 0.06Oe (5 A/m) for the as-prepared sample, up to ca. 1.26 Oe (100 A/m) for the conventional annealed microwire at the highest *T_ann_* of 400 °C.

The effect of conventional annealing on HLs of Co-rich microwires with low-negative magnetostriction *λ_s_* has already been studied [23]. Conventional annealing is expected to produce magnetic hardening, along with an increment in the magnetization of the metallic nucleus as evinced in the linear-to-rectangular evolution of the HLs in Figure 1a as *T_ann_* rises. The magnetic hardening is explained through internal stresses relaxation that brings about circumferential domain structure along the microwire. This hardening could also be the result of either a growth of inner axially magnetized domains [21,26,38], or a variation in the magnetostriction value [39,40], or even a sign change in the magnetostriction coefficient [23,40,41].

Hence, conventional annealing cannot be considered the best post-processing treatment to improve magnetic softness of Co-based microwires. In this regard, stress-annealing counteracts the magnetic hardening experienced by the microwire on conventional annealing. This effect is plainly visible in the insets of Figure 1b,c, where two characteristic stress-annealing temperatures are represented, *T_ann_* = 300 and 350 °C, respectively. There, the coercive field, *H_c_*, is reduced while achieving a magnetic softening, which is more clearly shown in Figure 2b. In addition, microwire soft magnetic properties can be affected by the stress-annealed conditions, i.e., *T_ann_*, *t_ann_* and *σ_m_* [27,33,40,41]. Figure 1c shows that the linear HLs typically observed for the as-prepared microwire are recovered at high enough *T_ann_* and *σ_m_* (in violet), but at the expense of *M_r_/M*_0_-values (where *M_r_* is the remanent magnetization). This decrease in the *M_r_/M*_0_ ratio results in a reduction of the GMI response as is observed below for the stress-annealed microwire at *T_ann_* = 300 °C and *σ_m_* = 472 MPa. These changes of magnetic properties result from an increase in circumferential magnetic anisotropy, which is induced by stress-annealing and becomes more significant when increasing *T_ann_*, *t_ann_* and *σ_m_* [33,38].

**Figure 3:**
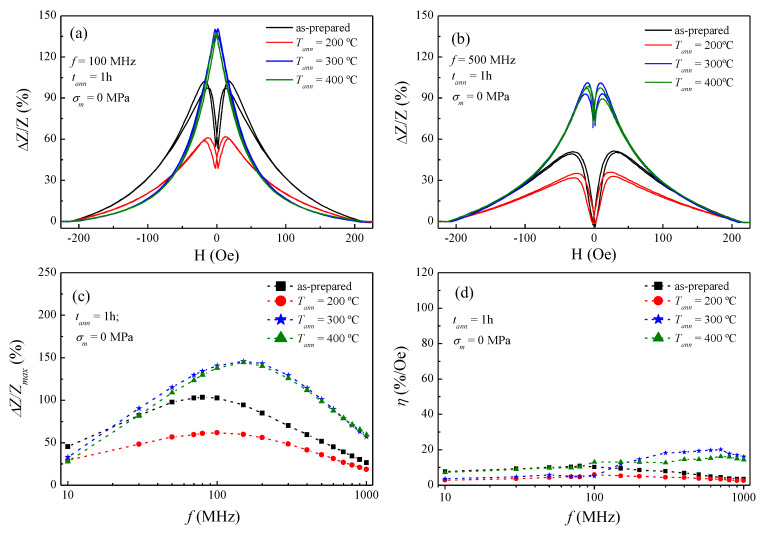
Field dependence of the GMI ratio for the as-prepared and annealed Co_69.2_Fe_3.6_Ni_1_B_12.5_Si_11_Mo_1.5_C_1.2_ microwires measured at representative frequencies: (**a**) 100 MHz and (**b**) 500 MHz; (**c**) frequency dependence of ΔZ/Z_max_, and (d) field sensitivity. Dashed-lines in (**c**) and (**d**) are a guide to the eye, while full-symbols denote experimental data.

In Figure 3 we confirm the dissimilar effect of conventional annealing on the GMI response depending on *T_ann_*. On the one hand, Figure 3a shows a reduction in the GMI ratio upon annealing at 200 °C, decreasing from 103% (for the as-prepared) down to 62% at 100 MHz. However, a clear improvement in the GMI effect is achieved when annealing at 300 °C, rising from 103% up to 141% at 100 MHz. Furthermore, despite the opposite effect of conventional annealing on the GMI response, it is noteworthy that in Figure 3c the maximum value of the Δ*Z/Z_max_* ratio is monotonously shifted to high-frequencies as the *T_ann_* rises. This characteristic frequency, hereinafter *f_char_*, is defined as the frequency at which the Δ*Z/Z_max_* ratio is maximal. In addition, in Figure 3d the field sensitivity displays minor differences in the frequency dependence between samples. Therefore, conventional annealing is revealed as an adequate treatment to improve the GMI response only at certain annealing conditions.

**Figure 4:**
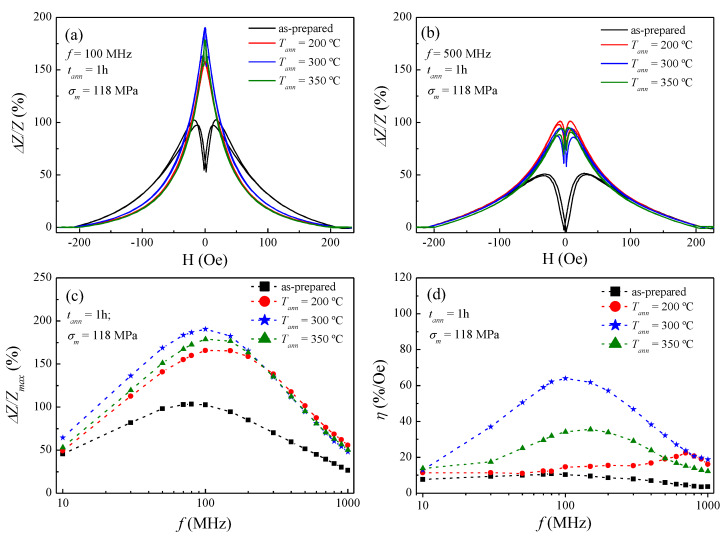
Field dependence of the GMI ratio for the as-prepared and 118 MPa stress-annealed Co_69.2_Fe_3.6_Ni_1_B_12.5_Si_11_Mo_1.5_C_1.2_ microwires measured at representative frequencies: (**a**) 100 MHz and (**b**) 500 MHz; (**c**) frequency dependence of ΔZ/Z_max_, and (**d**) field sensitivity. Dashed-lines in (**c**,**d**) are a guide to the eye, while full-symbols denote experimental data.

With regard to the effect of stress-annealing on the GMI response, in Figure 4 it is clearly appreciated as a significant GMI improvement as compared to conventional annealing for all stress-annealed microwires. For example, the measured Δ*Z/Z* ratio of the as-prepared microwire is equal to 103% at 100 MHz, whereas an increase up to 166% is found in the stress-annealed microwire at 200 °C and 118 MPa (see Figure 4a). By contrast, a reduction in the Δ*Z/Z* ratio down to 62% is observed in Figure 3a for the conventional annealed microwire at 200 °C. The improvement in the GMI response clearly is stated in Figure 4c for the whole frequency range (up to 1000 MHz). Moreover, Figure 4c shows a slight increase in *f_char_* up to ca. 150 MHz for the Δ*Z/Z_max_* of the stress-annealed microwires, in comparison with the as-prepared microwire where the maximum is centred at ca. 80 MHz. Figure 4d draws a clear improvement in the field sensitivity for the stress-annealed samples, in contrast to conventional annealing. Particularly, at 100 MHz, the field sensitivity rises from 10%/Oe for the as-prepared, up to *η* = 64%/Oe when stress-annealing at 300 °C and 118 MPa. However, the field sensitivity obtained for the three stress-annealed samples is quite similar in the frequency range 500 ≤ *f* ≤ 1000 MHz. In fact, they follow the same tendency as those shown in Figure 4c for the frequency dependence of Δ*Z/Z_max_*. In summary, stress-annealing arises here as the suitable technique to enhance simultaneously the GMI ratio and field sensitivity.

**Figure 5:**
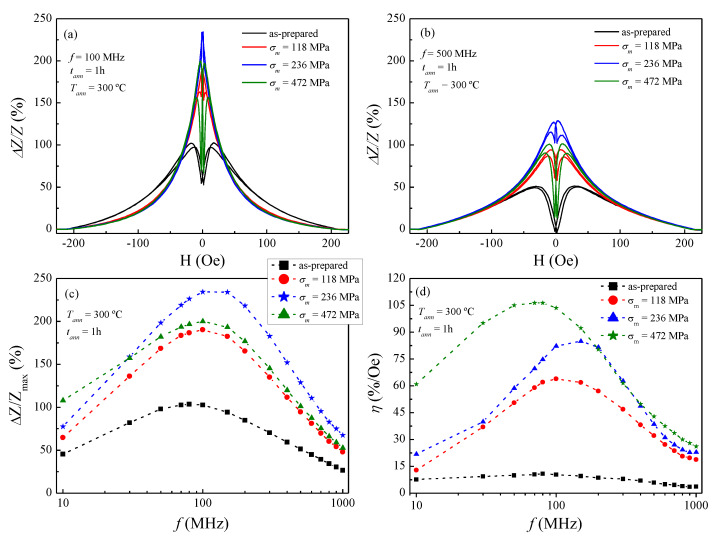
Field dependence of the GMI ratio for the as-prepared and stress-annealed Co_69.2_Fe_3.6_Ni_1_B_12.5_Si_11_Mo_1.5_C_1.2_ microwires measured at 300 °C: (**a**) 100 MHz and (**b**) 500 MHz; (**c**) frequency dependence of ΔZ/Z_max_, and (**d**) field sensitivity. Dashed-lines in (**c**,**d**) are a guide to the eye, while full-symbols denote experimental data.

In this line, the 118 MPa stress-annealed microwire at 300 °C presents the highest GMI response and field sensitivity, as show in Figure 4c,d, respectively. For this reason, it was resolved to make a more detailed investigation of the stress-annealing effect under different applied stresses, but at a fixed *T_ann_* of 300 °C (see Figure 5). It is worth noticing the remarkable improvement of both the GMI effect and field sensitivity, though at different stress-annealing treatments. Figure 5c shows the positive evolution of Δ*Z/Z_max_* as the applied stress *σ_m_* rises. In fact, this positive change is even more evident in the field sensitivity response (see Figure 5d). Specifically, at 100 MHz the GMI ratio improves up to 234% for an applied stress of 236 MPa, in contrast to the 103% for the as-prepared sample. On the other hand, the field sensitivity is enhanced up to 104%/Oe for the 472 MPa stress-annealed microwire, while the as-prepared exhibits a poor 10%/Oe. Therefore, the field sensitivity improves as the applied stress rises (see Figure 5d) within the following range 10 MHz ≤ *f* ≤ 200 MHz. This frequency range is the preferred for sensors applications because of better signal to noise features and hence lower price of electronic circuits, allowing easier processing of the electronic signals [17]. In short, this makes our stress-annealed sample a suitable material for sensor applications [9]. It is worth noting that for other families of thicker Co-rich wires (d = 120 μm) prepared by the in-rotating water technique a *f_char_* < 1 MHz has been reported [42].

All the experimental results reported here could be described considering that the origin of the GMI effect is directly connected to the skin depth (ac frequency *f*) and the circumferential magnetic permeability (through the external dc magnetic field *H_dc_*, ac current, and induced anisotropies). Consequently, a good understanding of both features is a requirement for high-performance soft magnetic materials [13]. In fact, it is well-known that both skin depth and circumferential permeability are interrelated, and in magnetic microwires of radius *a* can be given as follows [24,42,43]:

(4) $$\delta_{m}=\frac{1}{\sqrt{{\pi \;\sigma \;\mu }_{\phi }\;f}}$$

where *σ* and *μ_ϕ_* are the electrical conductivity and circumferential magnetic permeability, respectively.

In view of this, we can tackle the GMI response by drawing a general description of skin effect and magnetic anisotropy. The frequency studied in this paper covers the frequency range from 10 to 1000 MHz, in which the skin depth *δ_m_* plays an important role. In panels (a) and (b) of Figure 3, Figure 4 and Figure 5, the GMI exhibits the expected double-peak behaviour for the as-prepared sample (in the whole frequency range), which results from the induced magnetoelastic anisotropy during the fabrication. By contrast, at 100 MHz a single-peak response is achieved by specific conventional annealing (at *T_ann_* ≥ 300 °C in Figure 3a) and stress-annealed samples (*σ_m_* ≤ 236 MPa in Figure 4a and Figure 5a, respectively). This is the result of vanishing induced magnetoelastic anisotropies during their fabrication. Moreover, in annealed and stress-annealed samples (at low *σ_m_*-values) the internal stresses’ relaxation contributes to the axial anisotropy, and therefore, GMI response tends to be single-peak. However, in stress-annealed samples, at sufficiently high *σ_m_*-values, the circumferential anisotropy becomes more relevant and double-peak behaviour is observed at 100 MHz. In addition, at 500 MHz all the treated samples exhibit double-peak behaviour. Once more, this is straightforward explained through the frequency dependence of the skin effect in magnetic microwires (see Equation (4)), while the influence of magnetoelastic anisotropies are taken into consideration. In this sense, at relatively low frequencies, it is assumed that the current flows through the whole ferromagnetic nucleus, i.e., the skin depth is comparable to the microwire radius, i.e., *δ_m_ ≈ a* [33,44]. However, as frequencies increase, the skin depth decreases and hence the current flows closer to the surface while inducing the circumferential magnetic anisotropy near the metallic nucleus surface, which in turn becomes more relevant. For that reason, it is expected a magnetic evolution from single to double-peak behaviour in the GMI response for some of the samples considered in this study.

**Figure 6:**
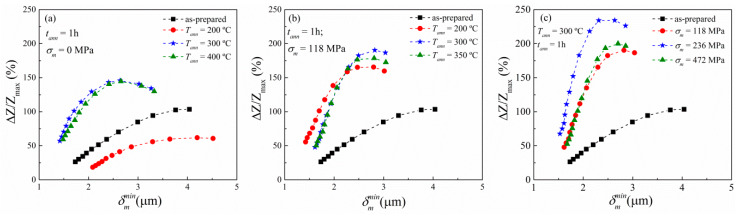
Minimum skin depth dependence of (∆*Z/Z*)_max_ for as-prepared and conventional annealed (**a**), as-prepared and stress-annealed at a fixed *σ_m_* of 118 MPa while varying the *T_ann_* (**b**), and at a fixed annealing temperature while varying the applied stress (**c**) for the Co_69.2_Fe_3.6_Ni_1_B_12.5_Si_11_Mo_1.5_C_1.2_ microwires. Dashed-lines are a guide to the eye, while full-symbols denote experimental data. The frequency dependence increases from right to left in contrast to panel (**c**) of Figure 3, Figure 4 and Figure 5.

Regarding the key role of the skin effect in the GMI response, where Z ~ 1/*δ_m_* [43,44], in Figure 6 we show the penetration skin depth dependence of (∆*Z/Z)_max_*. The skin depth *δ_m_* has been estimated through Equation (5) by considering that the real component of the measured impedance stems from the variations of the effective surface where the ac-current flows as a result of the skin effect [44,45,46]. This approach connects *δ_m_* with the ratio R_DC_/R_AC_, as follows:

(5) $$\delta_{m}=a\left[1-{\left(1-\frac{R_{DC}}{R_{AC}}\right)}^{1/2}\right]$$

where R_DC_ is the dc-resistance of the wire, and R_AC_ is the real component of the measured impedance at a given frequency as a function of the axially applied dc-field, and *a* is the wire radius. The minimum skin depth $\delta_{m}^{min}$ represented in Figure 6 is obtained from Equation (5) for each frequency in all samples.

Although, the (∆Z/Z)_max_ represented here redraws the same tendency as in the panel (c) of Figure 3, Figure 4 and Figure 5, that is, the increment (or diminishing for the conventional annealed sample at 200 °C) in the GMI effect depending on the treatment. In Figure 6 the treatment effect on the GMI response is more clearly evident. As a general rule of thumb, the treatment efficiency is directly related to the reduction of the $\delta_{m}^{min}$ while the GMI effect is improved. Thus, the skin depth not only gives a direct hint of the (positive/negative) influence of the treatment in the GMI response but also delimits the range of efficacy.

As mentioned above, the GMI response relies on the skin effect. Moreover, this effect only becomes relevant when $\delta_{m}≲a$, that is, in the intermediate frequency range where the maximal (Δ*Z/Z)_max_* is expected to occur [13,25]. Bear in mind that *f_char_* is defined as the frequency at which the GMI response is maximal. Taking this into consideration along with Equation (4), we obtain the following qualitative expression for the *f_char_* [43,44]:

(6) $$f_{char}=\frac{1}{{\pi \;\mu }_{\phi }{\;\sigma \;a}^{2}}$$

From Equation (6) it is inferred that the higher the circumferential magnetic permeability, the lower the *f_char_*. This is experimentally evinced in panel (c) of Figure 3, Figure 4 and Figure 5. Specifically, in Figure 3c the as-prepared sample exhibits the *f_char_* at 80 MHz, whereas the conventional annealed samples show the maximal GMI response at a higher *f_char_*, i.e., 100 MHz for the *T_ann_* at 200 °C and 150 MHz for the *T_ann_* samples at 300 and 400 °C. Similarly, Figure 4c and Figure 5c display the same tendency for the *f_char_.* In this sense, all the treated samples presented in this work show an increase in the *f_char_* as a result of the gradual internal-stresses relaxation that contributes to the axial anisotropy, but at the expense of the circumferential anisotropy.

Field sensitivity is represented in panel (d) of Figure 3, Figure 4 and Figure 5. There it is noticed the positive effect the stress-annealing on field sensitivity in Figure 5d. This could be derived either from the induced magnetoelastic anisotropy upon stress-annealing, or even from a change in the magnetostriction coefficient. Furthermore, Figure 4d shows an improvement in the sensitivity as the *T_ann_* rises up to a critical value of 300 °C. Above this temperature, the sensitivity is reduced. We surmise this counterbalance effect as a consequence of inner stress relaxation, i.e., the annealing above 300 °C vanish the magnetoelastic anisotropy in the microwire, which in turn is negatively reflected in both GMI response and field sensitivity.

Finally, microwires with different chemical composition and internal stresses (to a great extent related to the thickness of the glass-coating [30,31]) must present distinct GMI performance. However, similar effects of conventional annealing on the magnetic properties of Co-rich microwires of different chemical composition have already been reported, i.e., a significant magnetic hardening in various Co-based microwires with vanishing λ_s_-values has been noticed upon applying a conventional annealing [32]. Such magnetic hardening negatively affects the GMI performance [26,27,32]. In the present case, we have identified the precise route that enhances the GMI response and field sensitivity in the studied microwire with a well-established chemical composition. We anticipate that experimental dependencies concerning Co-rich microwires will be disclosed in the near future.

## 4. Conclusions

In summary, we have performed a comprehensive investigation of the GMI response and the field sensitivity by modifying the magnetoelastic anisotropies through different thermal treatments (conventional and stress-annealing) on the ferromagnetic amorphous Co_69.2_Fe_3.6_Ni_1_B_12.5_Si_11_Mo_1.5_C_1.2_ glass-coated microwire. On the one hand, the findings reported reveal stress-annealing as the suitable technique to improve simultaneous and remarkably the GMI effect and the field sensitivity. In this sense, it is observed a maximum GMI response of 234% for the exciting current frequency of 100 MHz and a maximum field sensitivity of 104%/Oe for the 300 °C stress-annealing sample at 236 and 472 MPa, respectively. Moreover, a significant frequency dependence of field sensitivity is attained in the stress-annealed samples. These results have been discussed in terms of the frequency dependence of skin depth, along with the magnetoelastic anisotropy modification. The presented outcomes can be used as a guide in further studies while deepening the knowledge to draw future lines in materials design. Hence, we evinced the stress-annealed Co_69.2_Fe_3.6_Ni_1_B_12.5_Si_11_Mo_1.5_C_1.2_ glass-coated microwire as a prospective material for sensor applications.
